# Phenylpropanoid Pathway Engineering: An Emerging Approach towards Plant Defense

**DOI:** 10.3390/pathogens9040312

**Published:** 2020-04-23

**Authors:** Vivek Yadav, Zhongyuan Wang, Chunhua Wei, Aduragbemi Amo, Bilal Ahmed, Xiaozhen Yang, Xian Zhang

**Affiliations:** 1 State Key Laboratory of Crop Stress Biology in Arid Areas, College of horticulture, Northwest A&F University, Xianyang 712100, China; vivekyadav@nwafu.edu.cn (V.Y.); zydx@nwafu.edu.cn (Z.W.); xjwend020405@nwafu.edu.cn (C.W.); bajwa1999@nwafu.edu.cn (B.A.); yxzh5186@126.com (X.Y.); 2 College of Agronomy, Northwest A&F University, Xianyang 712100, China; amoaristotle@nwafu.edu.cn

**Keywords:** phenylpropanoid pathway, plant defense, lignin, monolignol pathway, broad spectrum resistance

## Abstract

Pathogens hitting the plant cell wall is the first impetus that triggers the phenylpropanoid pathway for plant defense. The phenylpropanoid pathway bifurcates into the production of an enormous array of compounds based on the few intermediates of the shikimate pathway in response to cell wall breaches by pathogens. The whole metabolomic pathway is a complex network regulated by multiple gene families and it exhibits refined regulatory mechanisms at the transcriptional, post-transcriptional, and post-translational levels. The pathway genes are involved in the production of anti-microbial compounds as well as signaling molecules. The engineering in the metabolic pathway has led to a new plant defense system of which various mechanisms have been proposed including salicylic acid and antimicrobial mediated compounds. In recent years, some key players like phenylalanine ammonia lyases (PALs) from the phenylpropanoid pathway are proposed to have broad spectrum disease resistance (BSR) without yield penalties. Now we have more evidence than ever, yet little understanding about the pathway-based genes that orchestrate rapid, coordinated induction of phenylpropanoid defenses in response to microbial attack. It is not astonishing that mutants of pathway regulator genes can show conflicting results. Therefore, precise engineering of the pathway is an interesting strategy to aim at profitably tailored plants. Here, this review portrays the current progress and challenges for phenylpropanoid pathway-based resistance from the current prospective to provide a deeper understanding.

## 1. Introduction

As the first cellular compartment encountered during pathogen attack, it is significant to assume the functions of cell wall in plant defense [1]. The plant cell wall, being a dynamic and complex structure, has emerged as an essential component to monitor the defense responses. It is composed of a complex mixture made up of an intricate network of hemicellulose, cellulose, and pectin with the secondary cell wall comprised of lignin units [2]. The conventional viewpoint of the plant cell wall being a passive barrier has advanced to a concept that regards the wall as a dynamic structure, regulating both inducible and constitutive defense mechanisms and as a source of signaling molecules that activate immune responses [3]. The coevolution of plants and pathogens cause a series of strategic developments in attack and defense mechanisms. Perceptions of cell wall related resistance reactions to endeavored pathogenic attacks were first made over a century ago. Breaching of the plant cell wall is the only way for pathogens to gain access to cell components. To overcome the wall barrier, pathogens use cell wall degrading enzymes that bring changes in wall glycans or hydrolyze the linkage between glycan moieties, and develop penetration-specific structures, that exert turgor pressure [4,5]. Plants have developed a multi-layered defense network to fight microbial pathogen infection. At the initial stage, prior to disease establishment, cell wall related defense can stop invading pathogens, thus, eliminating the possible use of defense responses such as hypersensitive response (HR) cell death. The initial response to the recognition of various groups of pathogens involves a dynamic augmentation of the cell wall by depositing cell wall appositions generally regarded as papillae. Pectin is one of the primary structures to be modified during pathogen intrusion and proof shows that post-synthetic changes in pectic polysaccharides influence the resistance of plants to pathogens [6,7]. Vogel et al. [8] speculated that adjustment in pectin alteration may bring the discharge of defense elicitor-dynamic fragments upon degeneration by powdery mildew hydrolytic enzymes. The cell wall is attached to the cell surface through cell wall biosynthetic machinery, sensory, and structural proteins [9]. The plant cell wall detecting system has been posited to incorporate molecular network monitoring for (I) osmo-recognition; (ii) mechano-recognition; (iii) cell wall damage (CWD) recognition; and (iv) wall-derived ligand–receptor (PRR) recognition [5]. All these sensing frameworks initiate signal transfers through protein kinases (PKs) or/and calcium-based signaling cascade, stimulating the production of certain phytohormones. Consequently, downstream genes are regulated for the versatile changes in cell wall synthesis and structure, which, in turn, initiate the immune responses [10,11]. Recently, it has been shown that plants have evolved complex stress tracking frameworks through cell wall integrity (CWI) support mechanism [12]. Some portion of such CWI monitoring frameworks depends on the perception of “danger” alert signs, which offer signaling segments and reactions with the immune pathways activated by non-self MAMPs. Overall, infection is restricted upon the perception of pathogen or microorganism associated molecular pattern (PAMPs or MAMPs) by pattern recognition receptors [13]. The breakdown of cell wall integrity can be understood basically in two ways i.e., the puncture process by mechanical force propelled by turgor pressure or enzymes that degrade the cell wall, or both [14]. Experimental proofs have been accumulated over the last few decades to support the cell wall response for plant defense [15]. 

Phenylpropanoid pathway providing the lignin-building monolignols is greatly triggered after the cell wall gets hit by pathogens [16]. The first line defense system has dynamically evolved into different mode of defense, including deposition of different glycans, callose [17,18], phenolics and antimicrobial substances, defense papillae formation [19], lignification [20,21,22], reactive oxygen species production, and depositions of phytoalexins [23]. Perturbation of the phenylpropanoid and lignin biosynthesis pathway becomes easy with the induction of mutations or genetic engineering, resulting in a shift in physical as well as metabolic functioning of the pathway. The gene families coding for enzymes involved in biosynthesis have significant effects on physiochemical properties of cell wall. For example, 12 genes putatively involved in phenylpropanoid and monolignol biogenesis pathways were upregulated [24], and expression of *CsCCR* was induced after a fungal infection [25]. Similarly, phenylalanine ammonia-lyase, (hydroxy)cinnamoyl CoA reductase and (hydroxy)cinnamyl alcohol dehydrogenase activities were enhanced after fungal infection in *Lilium usitatissimum* [26]. Transcription factors (TFs) are believed to have an essential function in transmitting pathogen-derived defense signals either to initiate or repress downstream defense gene expression [27,28]. To accomplish all these, transcriptional regulator structure is a significant node to adjust the trade-off among development and defense for ideal distribution of resources and plant survival. In addition, apart from the intermediate pathway genes, there is the utilization of transcription factors for manipulating lignin contents and composition of vascular cell walls in various plant species [29,30,31,32]. The complex transcriptional regulation of lignification is a subject of great concern because it has the potential of being utilized in producing low or high-lignin biomass feedstocks as well as developing resistance in crops to diseases. The regulation of defense related transcription factors including MYB, bZIP, HD-zip, and WRKY are accepted by researchers due to its easy binding site of AC-element, which is commonly found in phenylpropanoid pathway genes [33]. Genes that encode TFs associated with plant resistance signaling are likewise transcriptionally controlled by pathogen challenge and treatment with defense elicitors. Instances of these are the jasmonic acid (JA)-enacted ERF1 and *AtMYC2* TFs that regulate JA reactions [27,28]. Thus, it is suggested that a potential strategy to recognize TFs that function in plant defense might be to initially distinguish TF genes that show altered transcript levels during the initial period of the defense response, coupled with the functional analysis of these candidate genes.

More recently, a link has been established for the role of key players from the phenylpropanoid pathway in broad spectrum disease resistance. A noteworthy example is the PAL genes which are found reliable for cell wall mediated immunity and involved in broad spectrum disease resistance. [34]. The race specific and quantitative resistance in plants provide either short term or incomplete protection against many diseases. The major objective for plant pathology is to develop the resistance without a compromise in yield of crop. Plants solely rely on innate immunity to perceive and ward off potential pathogens [35]. The progress in resistance (R) genes is limited to the race specific resistance to adapted pathogens and their durability is a major concern for researchers. The multiple gene incorporation is technically tedious and a time-consuming job, and it could compromise the production and productivity of the crop. 

However, the protective function and mechanisms, contributing to this immune homeostasis in different species are still uncovered and detailed information is required to validate the hypothesis to consider key players for a broad spectrum disease resistance (BSR) based approach to reduce yield penalties.

The objective of this review is to provide detailed information about the progress and challenges in understanding the role of the major genes and transcriptome network probably involved in the biosynthesis pathway of the lignin/phenylpropanoid pathway for plant disease resistance mechanism and also provide brief information about the current hypothesis as well as open questions for future prospects in this direction. Additionally, an attempt has been made to bring all the information together for pathway based emerging players in disease response and possible models have been provided for various resistance mechanisms.

## 2. Phenylpropanoid Pathways—Biochemistry to Genetics

**Figure 1:**
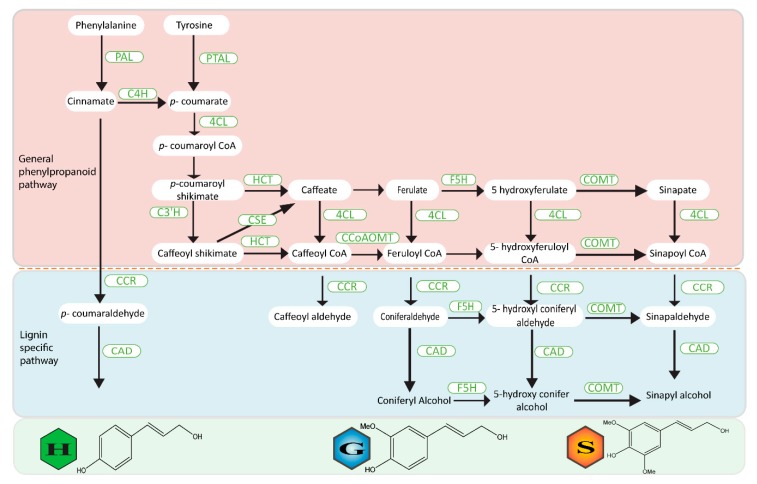
Phenylpropanoid and monolignol biosynthesis pathway in higher plants. Enzymes: PAL, phenylalanine ammonia lyase; PTAL, phenylalanine tyrosine ammonia lyase; C4H, cinnamic acid4 hydroxylase; CAD, (hydroxy)cinnamyl alcohol dehydrogenase; HCT, hydroxycinnamoyl-CoA:shikimate/quinate hydroxycinnamoyltransferase; C3′H, p-coumaroyl shikimate3′ hydroxylase; 4CL, 4-hydroxycinnamoyl-CoA ligase; CSE, caffeoyl shikimate esterase; CCR, cinnamoyl-CoA reductase; F5H, coniferaldehyde/ferulate5 hydroxylase; COMT, caffeic acid/5-hydroxyferulic acid O-methyltransferase; CCoAOMT, caffeoyl-CoAO methyltransferase.

The phenylpropanoid pathway has been constantly redrawn in recent decades, but now appears to be well established (Figure 1) [36,37,38]. The lignin biosynthesis pathway can be divided into two major groups: (1) the general phenylpropanoid pathway, comprising of several conversion steps from Phenylamine to feruloyl-CoA and (2) the monolignol-specified pathway that includes conversion of feruloyl-CoA to different monolignols [37]. Phenylalanine (Phe) acts as the initial substrate in almost all the species except grasses [39]. The pathway is a combination of deamination, methylation, hydroxylation, and two consecutive monolignol side chain reduction. These reactions are mediated by at least 11 enzymes including phenylalanine ammonia lyase (PAL), cinnamate 4-hydroxylase (C4H), (hydroxy)cinnamyl alcohol dehydrogenase (CAD), caffeic acid (5-hydroxyconiferaldehyde) O-methyltransferase (COMT), caffeoyl CoA O-methyltransferase (CCoAOMT) (hydroxy)cinnamoyl CoA reductase (CCR), 4-coumarate: CoA ligase (4CL), hydroxycinnamoyl-CoA shikimate/quinate hydroxycinnamoyl transferase (HCT), p-coumarate3-hydroxylase (C3H), caffeoyl shikimate esterase (CSE) (Figure 1). 

The conversion activity of PAL and ammonia lyase of Phe into cinnamic acid and *p*-coumaric acid kick start the general phenylpropanoid pathway [36]. The biosynthesis of hydrocyanic alcohols is triggered by the reduction of carboxylic acid and, for coniferyl and sinapyl alcohols, the aromatic ring is methoxylated (Figure 1). This part commonly involves PAL, C4H, and 4CL and is sometimes referred to as a general phenylpropanoid pathway. Hydroxylation and O-methylation of the aromatic ring of precursors at the level of hydroxycinnamic acids, with p-coumaric acid, ferulic acid, and sinapic acid are subsequently activated to their respective CoA thioesters and then reduced by CCR and CAD to yield *p*-coumaryl alcohol, coniferyl alcohol, and sinapyl alcohol, respectively [40]. The enzyme CCR helps in reducing hydroxycinnamoyl CoA thioesters to hydroxycinnam aldehydes and then with the aid of CAD enzyme is eventually converted to hydroxycinnamyl alcohols [41]. Overall, lignin G units and caffeoyl alcohol are generated by enzymes HCT, CCR, C3H, and CAD. An alternate branched pathway leads to sinapyl alcohol and S units via the action of F5H, COMT, and CAD (Figure 1). The CSE enzyme has recently been added into the well-established lignin biosynthesis pathway due to its action on caffeoyl shikimate [42]. Apart from this general pathway, a new study in a model grass carried out by Barros et al. [39] proposed a new monolignol biosynthesis process. The tyrosine pathway is a shortcut to producing the monolignol by eliminating the steps of catalyzation by C4H. In this process, the tyrosine is converted into *p*-coumarate by a bifunctional phenylalanine and tyrosine ammonialyase (PTAL).

## 3. Phenylpropanoid and Lignin Pathway associated Genes in Plant Defense

The phenylpropanoid pathway entails the biogenesis of various number of phenolics polymers and lignin, and these compounds contribute to various disease resistance mechanisms [43,44,45,46]. The intermediate metabolites and phenylpropanoid compounds produced during lignin biosynthesis pathway display antimicrobial activities and are involved in plant defense. The genes involved in shikimate pathways are known and facts about their response regarding initial access to cell walls are demonstrated in some studies [36,47]. Lignin is most abundant ubiquitous biopolymer after cellulose and accounts for 30% of organic carbon in the biosphere [48]. The number of genes from phenylpropanoid pathways are found to be highly expressed in biotic and abiotic stress conditions, resulting in an increase in the accumulation of corresponding enzyme and boost in enzymatic activities [49,50]. Sinapyl alcohol and coniferyl monolignol are the major building blocks of lignin and they generate syringyl (S) and guaiacyl (G) units of lignin polymers, respectively [51,52]. The minor monolignol unit which is deposited more in monocots is the p-hydroxyphenyl (H) unit derived from p-hydroxyphenyl. Lignin plays various roles in different kinds of stress conditions. The flux disruption in the phenylpropanoid pathway could be the reason for some resistance mechanism because this pathway generates various compounds (phenolic phytoalexins, stilbenes, coumarins) [53,54,55] besides monolignols which have also been implicated in plant defense [56]. Disruption in the gene families in lignin pathway can bring some unpredictable responses towards the disease resistance and the amount and ratio of lignin can influence its physico-chemical properties. For example, a shift in S/G lignin subunit ratio does not influence any morphological changes, but changes in the sequestration of molecules involved in defense signal in the cell wall matrix, and hence, the transcript levels of genes responsible for response to stress [57]. Previous studies indicated that some alternations were positively regulated towards the resistance. The disease response was studied in *Arabidopsis thaliana* by either silencing or deletion of functional mutations of genes in regulatory pathways. These studies were mainly focused on some gene families such as PAL, F5H. C4H, COMT, 4CL, CCR, CAD, HCT, C3H, CSE, and CCoAOMT. Some recent results related to the major genes in monolignol pathways for defense are listed in Table 1. After analyzing the findings, it can be concluded that alteration in pathway genes can cause a shift in the part of metabolic flux with monolignol units and, in this way, many things can be altered [58]. It is very hard to predict the function of genes/gene families against disease response because the regulation is mostly dependent on the crop and disease, moreover, the pathogen type and crop are also major factors which influence the mechanism. Overall, responses also revealed that a single gene disruption can cause different effects on crops and diseases [59]. Lignin pathway alteration may influence the response of pathogens through either increased susceptibility [37] or, paradoxically, increased resistance [59]. The latter may perhaps be related to the trigger of multiple defense pathways (detailed discussion in Section 5.).

## 4. Transcriptional Regulation for Phenylpropanoid Pathways

It is generally assumed that transcriptionally activated genes involved in varied biosynthetic pathways result in the emergence of phenylpropanoids metabolites during the response of plants to infection (Table 2). However, it must be noted that this assumption, in most cases, is deduced from measuring the stable state transcript levels. The measurement of the stable state transcript level does not differentiate between a rise in mRNA stability and increased transcription [56]. Nonetheless, several studies have revealed a direct link between the induction of infection and a surge in the rate of transcription of phenylpropanoids pathway genes, which was estimated using nuclear transcript run-on assays [81,82], and there is an appreciable interest in describing various transcription factors for synchronizing upregulation of pathways involved in defense response.

Many prediction methods including cis-regulatory elements of monolignol biosynthesis pathway genes have helped reveal the mystery of transcriptional regulation of phenylpropanoid pathways. Promoter and electrophoretic mobility shift assay (EMSA) analysis have supported the prediction and concrete results for complex transcriptional networks. There is a regulatory mechanism exhibited by the phenylpropanoid pathway at the transcriptional, post-transcriptional, and post-translational levels [83]. The promoters of genes in the lignin biosynthesis pathways have specific binding locations for MYB, LIM, ERF, and KNOX transcriptional factors [84,85].

The key findings of the existence of AC elements in the promoters from various studies are widely accepted. Deep functional analysis of promoter region of the genes in the phenylpropanoid pathway revealed their modular organization and identified AC elements as central coordinator elements. Studies show that AC-rich elements correlating with the MYB transcription factor-binding motif are essential for coordinated pathway genes [30,86,87]. It has been revealed that that the promoter region analysis of most of the phenylpropanoid pathway genes like PAL, 4CL, C3H, CCoAOMT, CCR, and CAD provides sufficient proof of the presence of AC elements. However, genes involved in S-lignin biosynthesis did not carry the AC-rich element and were not regulated by lignin specific transcriptome factors. Instead, they had a good linkage with NST3 (SND1) [88], which regulates MYB factors [89].

**Figure 2:**
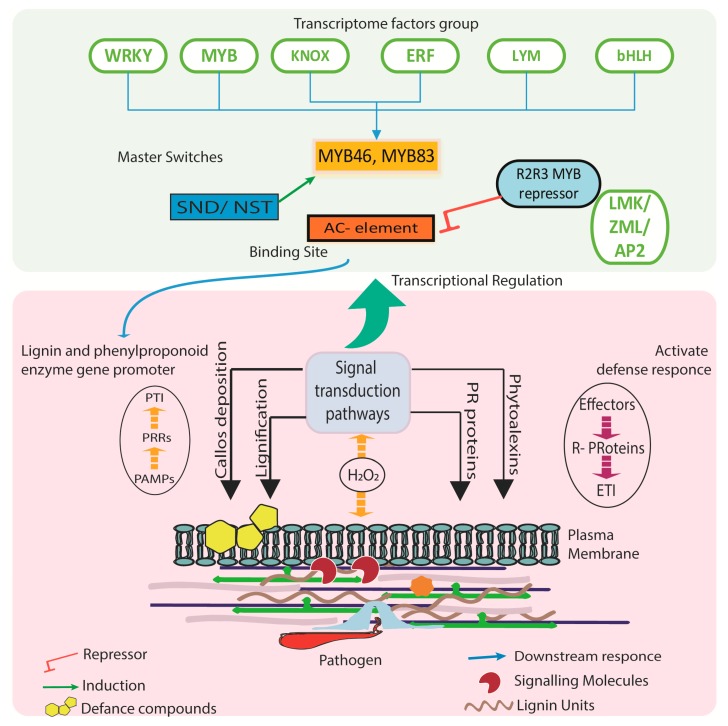
Summary of defense related transcription factors of phenylpropanoid pathway. MYB activators bind to AC elements in enzyme gene promoters and activate gene expression. Constitutive response mechanism of transcription factor involved in phenylpropanoid and lignin biosynthesis pathway.

The role of MYB family is highly linked as an activator in the monolignol biosynthesis pathway. MYB46 and its homologous MYB83 were discovered to not only actively participate in lignin biosynthesis but redundantly activate the formation of secondary cell wall [90,91]. The proposed model to date shows NST genes and MYB46/MYB83 can be activated by the biogenesis of secondary cells, and in depth NST genes are directly linked to MYB46 and its homologous genes. The first active transcriptional factors from MYBs were identified in differentiating xylem. Patazlaff et al. [92] conducted an experiment on *Pinus taeda* lignifying tissue and discovered *PtMYB4*. In tobacco plant, the overexpression of *PtMYB4* resulted in an increased expression of C3H, COMT, CCR, CCoAOMT, and CAD with the expression of 4CL and C4H genes reportedly unaffected but resulted in a decline in the expression of PAL genes. Moreover, the case was a little different in transgenic tobacco plants. Transgenic tomato plants did not respond to the PAL, C4H genes but there was a significant increase in expression of the monolignol-specific coding genes. Some transcriptional factors are found to act as repressors of lignin biogenesis [93,94,95,96]. The current knowledge about the repressors shows that the discovered repressors belong to a subgroup of the R2R3-MYB transcriptional factor family, which is the most common MYB transcription factor in plants and can be categorized into a general phenylpropanoid and lignin group and a flavonoid group. However, bHLH-binding domain is common in both types of repressors [97]. The overall mechanism of transcriptome regulators involved in lignin-based defense are illustrated in Figure 2. 

## 5. Mechanism of Player Genes in Defense Response

The compounds generated by the phenylpropanoid pathways are one of the important reasons for its defensive functions. The genes in the phenylpropanoid pathway code for various compounds. Defensive properties are not only restricted to any class of phenylpropanoid, but are regulated by hydroxycinnamic acids and monolignols through various complex spectra of mechanisms. The genes responsible for the defensive compounds have been cloned in many crops. The biosynthesis pathway is governed by gene families, but the function of specific members is yet to be determined. The set of genes involved in biosynthesis pathways have some mechanisms of defense, including the role from lignification, utilization of secondary metabolites to elicitors and defense singling regulations. Important defense mechanisms are discussed below: 

### 5.1. Lignification

The basal defense mechanism starts with the lignification of the cell wall innate response to plant diseases apart from its contribution to structural integrity [125]. Composition of lignin in the woody cell wall biomass contributes to 30% and this aromatic polymer has multidimensional functions. The lignification process includes the deposition of lignin into intercellular voids between cell wall polymers [126]. The process of lignification being a non-random process is governed by many factors. However, as compared to biosynthesis, the processes of monolignol lignification and polymerization remain largely unclear. The build-up of lignin or lignin biosynthesis phenolic compounds are known to counter pathogenic diseases. From the aspect of plant development, lignification is the last process of secondary cell wall (SCW) biosynthesis. Lignification increases the resistance capabilities of the cell wall by generating mechanical pressure during fungal attack and penetration [127]. Lignification can act in several ways, such as establishment of a mechanical barrier to pathogen attack, chemical modification for wall-degrading enzymes, increasing resistance to wall by diffusion of toxins, production of toxic precursors, and free radical production. In addition, lignification may restrict pathogen invasion process through protecting the cell wall from degradation by pathogen secreted enzymes. [83]. 

Heterogeneity and nature of monolignol linkages make it difficult to degrade and is reported to function as a barrier to limit the colony development or penetration process by pathogens, representing the basal defense mechanism. [44,128]. After an immediate hit on the plant cell wall by pathogens, the process of lignin deposition is initiated [129]. Biochemical analysis indicates that lignified material is a major component of cell wall appositions [130,131]. 

Furthermore, lignin-based compounds may hamper the multiplication and movement of pathogens [37,132]. Mandal et al. [133] discovered a higher concentration of lignin in tomato genotypes that were resistant to bacteria. The expression of lignin related genes was recorded to be influenced directly by pathogen infection processes. Expression of *AtCAD5* was recorded higher in roots with lignification during the pathogen infection [134]. DIRs (dirigent genes) were involved in lignin deposition in *Capsicum annuum* L. [131] and *Gossypium hirsutum* during pathogen infection [135]. Additionally, the HCT gene was upregulated in maize and binding with NLR Rpl protein caused an increase in lignin accumulation [136] and the decreased accumulation of lignin resulted in an increase in sensitivity against rice blast [137]. The difference in level of lignin and other metabolites of phenylpropanoids has been suggested to be linked with the CCoAOMT gene in maize for resistance to multiple pathogens [71]. 

### 5.2. Coumarins, Flavonoid, Phytoalexins Naringenin (Metabolites)

**Figure 3:**
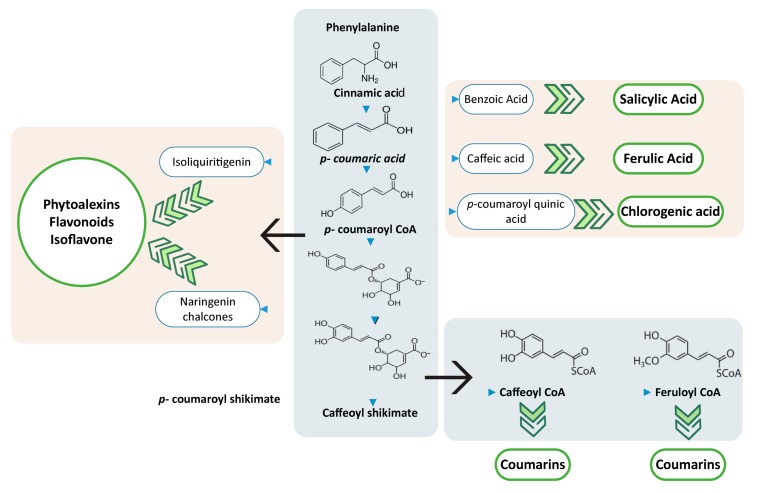
Biosynthesis of plant defense related compounds from phenylpropanoid and monolignol pathways.

The phenylpropanoid pathway is generally related with the production of flavonoids, monolignols, phenolic acids, stilbenes, phytoalexins, and coumarins and these compounds (Figure 3) are directly and indirectly involved in plant development and disease response [138]. These secondary metabolites have their specific mechanism to counter the disease response [139]. In recent decades, the responses against disease have been studied to understand the critical regulations and mechanism. 

#### 5.2.1. Coumarins

Plant derived compounds which are involved in plant disease defense mechanisms are either phytoanticipins or phytoalexins. In recent decades, coumarins got the attention of plant scientists for their role as phytoanticipins or phytoalexins in the plant defense system [140]. Coumarins are a group of compounds present in wide range of plants and derived from phenylpropanoid pathway. The phenylpropanoid pathway gene CCoAOMT was recorded to be involved not only in synthesis of lignin but also in production of coumarins. Knock out of the gene that encodes for CCoAOMT1 led to a decline of coumarin scopoletin in *A. thaliana* roots [141]. Coumarins are also known to be iron mobilizing compounds. Research in different plants supported the fact that coumarin accumulation initiates in plant tissue during plant–pathogen interaction processes. Scopoletin and its glucosylated form scopolin are the general coumarins reported to have antimicrobial and antioxidative properties in disease resistance [142,143,144]. Coumarins may also be engaged in the accumulation of reactive oxygen intermediates (ROI), one of the important processes during the early stage of cell death. The antisense plants of tobacco were used for coumarin response and enormous ROI build-up relative to the control plant was recorded [144]. The accumulation of coumarins were reported higher during the infection of tobacco mosaic virus (TMV) in tobacco leaves [145]. A similar observation was recorded in rubber plant after *Phytophthora palmivor* infection [146]. Some more relevant recent studies for role of coumarin in plant–disease interactions are listed in Table 3.

#### 5.2.2. Flavonoids

Plants have ability to synthesize many important compounds which prominently function to protect the plants [155]. The main types of secondary metabolites namely phenolics, flavonoids, terpenes, and nitrogen/sulphur containing compounds are generated in plant systems. Flavonoids are important compounds derived from phenylpropanoid pathway in plants and they are extensively utilized to treat microbial diseases. The defense related flavonoids can be divided into two groups “preformed” and “induced” on the basis of their mode of action. The “induced” compounds are synthesized after stress and “performed” flavonoids are synthesized during general development of plants [155]. It is also known that flavonoids have potential to target multiple sites in bacterial cells [156,157]. While some flavonoids restrict the cytoplasmic function, sophotaflavanone and catechins are reported to have a direct impact in damaging the bacterial membrane [158]. For example, flavanone phytoalexin, sakuranetin has been reported as important for rice blast disease resistance [159].

### 5.3. SA-Mediated Resistance

Beside the mechanism discussed above, it has been proposed that plant infestation initiates a rise in the activity of PAL, a key enzyme from the phenylpropanoid pathway. In plants, it is believed that SA formation is attained either through the isochorismate synthase (ICS) or the PAL catalyzed steps. SA is a key player in conferring resistance to disease and pathogens in various crops. SA dependent signaling regulates the activation of plant defense thereby warding off microbial pathogen attacks [160,161,162] and as such making SA a key component of the defense of plants to various pathogens. SA mediation of plant defenses against pathogens could be the most promising avenue used by plants from the phenylpropanoid pathway by activating the precursor enzymes of SA directly or indirectly. For example, in soybean, Shine et al. [64] revealed that the aftermath of PAL knock-down resulted in SA biosynthesis shutdown and abrogation of pathogen resistance. In maize, PAL contribution to resistance against sugarcane mosaic virus is highly linked to the positive regulation of salicylic acid build-up [163]. Similarly, in *A. thaliana*, the production of SA precursors was a major function of PAL during *Peronodpora parasitica* infection. The effect of PAL2.1 (*GmPAL 2.1*) was checked on soybean after *Phytophthora sojae* infection and it was found that the SA level was regulated by the PAL genes [164]. Apart from the direct link with disease resistance and involvement of phenylpropanoid pathways with SA mechanism, studies carried out by Peng et al. [165] showed a rapid build-up of a high level of SA after tomato leaves were exposed to cotton bollwarm, a kind of chewing pest and potential carrier of disease. Furthermore, Verticillium wilt, a serious cotton disease was found to be associated with WATs (Walls Are Thin gene) for lignin deposition resistance modulated by SA biosynthesis [166]. Duan et al. [167] revealed a dramatic surge in PAL expression after small brown plant hopper (SBPH) sucking, leading to SA synthesis and a noticeable alternation in SA content in rice. It suggests the SA mediated signaling pathway is one of the crucial defense mechanisms in conferring resistance in rice.

### 5.4. Signaling and Elicitor Based Pathway

In comparison with other resistance mechanisms, elicitor mediated signaling is of great importance. The whole sensing system relays via protein kinase or calcium depended signaling systems. It has direct relation with activation of phytohormone pathways, which in turn govern the activation of immune responses. For example, in a yeast-based experiment system, the involvement of defense regulating *Rac* family was found to interact with *OsCCR*, which is a lignin pathway regulator [168]. In a different case study, NH1-mediated resistance for *Xanthomonas* was governed by the CCR-like gene and SNL6, which have an effect on lignin content of rice [169].

## 6. Virulence Pathogen Regulates Phenylpropanoid Pathway

Generally, after penetration, pathogens exploit the metabolism of host cell penetration of the cell wall [138] and virulence pathogens also adopt some mechanisms to suppress host defenses for promoting their growth. Many pathogens use effector proteins to modulate host immunity and physiology [170]. Specifically, for fungi, various means have been harnessed by pathogens, involving induction of molecules which inhibit defense-inducing molecules in hosts [171,172]. The effectors from the AvrE family were found to suppress SA pathway, callose deposition, and PR1 expression [173,174]. The common means adopted by plant in defense against fungal pathogen is by depositing phenolic compounds in the cell wall [175]. Phenylpropanoid metabolism results in the generation and production of different varieties of metabolites [176] and turns out to be the major target and focus of phytopathogenic bacteria and various pathogens of plants. For example, *P. syringae* disturbed the expression of major enzymes of the phenylpropanoids pathways and also downregulated the ability of *A. thaliana* to photosynthesize [177]. In maize, *Ustilago maydis*, a biotrophic fungus and causal agent of smut disease, tends to deploy effector proteins that repress the production of SA [178] and similarly, during bacterial wilt of tomato PopS effectors were utilized by *Ralstonia solanacerum* to overcome SA mediated defense [179]. It was also revealed that *P. syringae* Type III Effector (T3E), HopI1 impedes SA production by attacking the chloroplast localized chaperone [180]. In similar fashion, Zhou et al. [181] revealed that variant of *P. syringae* T3E, HopZ1b, represses the phytoalexins precursor production by targeting a soybean (*Glycine max*) 2-hydroxyisoflavanone dehydratase. In apple, Venisse et al. [182] showed that fire blight pathogen is capable, specifically in susceptible genotypes, to precisely block gene expression that encodes enzymes of the pathway of phenylpropanoids metabolism. This ability is linked to the bacterial functional Hrp-secretory apparatus. A similar finding was reported for the AvrE superfamily of T3E involved in suppression of plant immunity [183]. Moreover, phenylpropanoid metabolism disruption was recorded in maize by the AvrE family T3E from *Pantoea stewartia* [184].

## 7. PALs: Emerging Key Players in Broad Spectrum Disease Resistance

Crops are prone to attack by different pathogens causing various diseases and so the quest to breed for crops tolerant to diseases has never been more urgent and tasking till now. Diseases lead to an overall yield loss of about 16% globally [185]. The utilization of a single R gene in the long run results in a resistant breakdown to the pathogen. Adopting the strategy of pyramiding R genes seems plausible but time consuming and technically demanding [35]. There is, therefore, a need to breed for crop varieties that confer broad spectrum resistance to pathogens. 

The vast majority of the phenylpropanoids have been shown to confer broad spectrum antimicrobial activity and also help plants defend against microbial diseases [55]. For example, in plants, SA plays the role of activating S key signals responsible for disease resistance in plants. Therefore, when the defense signaling pathway against pathogens is activated, this would result in the accumulation of SA. In order to achieve systemic resistance, conferring resistance to a broad spectrum of pathogens, the movement of methylated derivates of SA from local infected tissue to proximal tissues initiates [186]. *Oryzae Ssativa* genome consists of nine *OsPAL* genes. Most of the *OsPAL* genes experience an upregulation during resistance interaction with *R. solani, M. oryzae*, and *X. oryzae*. Eight of the *OsPAL* genes are co-localized with QTLs responsible for resistant to multiple pathogens in rice. However, *OsPAL9* shows no association with any QTL for resistance. For example, *OsPAL1, OsPAL2, OsPAL3*, and *OsPAL4* are within the bounds of three QTLs associated with resistant for sheath blight qSB-2, qSBR2-2 [187], *OsPAL5,* and *OsPAL6* are within the bounds of a QTL linked with bacterial blight resistance [188], and a single QTL associated with rice blast resistance, CQAC2 [189]. Using IR64 genetic background, Tonnessen et al. [190] identified *OsPAL4*, a mutant line bearing a deletion in the *OsPAL4* gene was linked to a rise in susceptibility to three pathogens, *X. oryzaepv. oryzae, R. solani*, and *M. oryzae*. This revealed that contribution of *OsPAL4* to broad spectrum resistance is achieved by the QTL located on chromosome no. 2, which is responsible for disease resistance in rice. In addition, *OsPAL4* mutant showed an increase in susceptibility to both *X. oryzaepv. Oryzae* and *M. oryzae* as compared to the wild type, confirming that *OsPAL4* mutant affects resistance to disease in a negative fashion through altering expression of other *OsPAL* genes, such as *OsPAL6* [190]. Duan L. et al. [167] used the wild-type Zhonghua 11 genetic background and found that *OsPAL6* is involved in the regulation of *M. oryzae* resistance invaded from roots by promoting phytoalexins and SA synthesis, which then turns out to be influential in JA- and ethylene-dependent signaling. Furthermore, beside the contribution of *OsPAL06*, it is likely that *OsPAL1, OsPAL2, OsPAL3, OsPAL4, OsPAL5, OsPAL7*, and *OsPAL8* participate in the rice—*M. oryzae* interactions. Following the rice yellow mottle virus infection, there was an increase in the synthetization of *OsPAL1* and *OsPAL2* proteins During rice–*Xanthomonas oryzae pv. oryzae* interactions, *OsPAL01* also showed distinctive expression [191,192,193]. These findings suggest that *OsPAL*s do not just play a part in reactions involving resistance but also play a role in susceptible reaction and diverse *OsPAL*s take part during various points of rice–pathogen interactions. It is suggested that *OsPAL* may play a key role in broad spectrum resistance. The adaptation of *M. oryzae* is carried out in such a way that its virulence proteins cannot be identified by the resistant gene product [194]. Since the functions of *OsPAL1* and *OsPAL6* in rice are downstream of defense-responsive pathogen species or race-nonspecific resistance, it can also be suggested that resistance mediated by PAL may be durable due to the fact that its product does not involve pathogen recognition [193,195]. 

The PAL gene family consists of three to four genes in pepper plant (*Capsicum annum*). The R gene-mediated and basal resistance to infection by *Xanthomonas campestris pv. vesicatoria* is contributed by the activity of PAL in pepper plants. A study carried out by Kim et al. [65] showed that the *CaPAL* gene is necessary for inducing SA-dependent defense signaling activities in plants [65]. This result suggests that in response to avirulent and virulent *Xanthomonas campestris pv. vesicatoria* infection, *CaPAL1*-silenced pepper plants displayed an increase in susceptibility. Likewise, in *A. thaliana*, there was an increase in resistance against *Hyaloperonospora arabidopsidis* and *Pseudomonas syringaepv. tomato* (Pst) and in plants with overexpression of *CaPAL1* suggesting SA build-up in the course of Pst infection was upregulated by the constitutive overexpression of *CaPAL1* in Arabidopsis. In a recent study, Shine et al. showed that SA functions in conferring resistance against oomycete pathogen *P. sojae* and bacterial pathogens *P. syringae* and the build-up of phenylalanine substrate was as a result of silencing *GmPAL* with ICS-catalyzed and PAL catalyzed reactions playing a combined role in soybean defense. 

The first report of PAL gene was done in 1961 and over a decade, the number of studies in different plants have shown the wide distribution of PAL gene families in higher plants, and is also reported in yeast and some bacteria [196]. The PAL gene family is a small gene family with 1–20 members in different plant species. In recent years, structure, evolution, and resistance mechanisms to biotic and abiotic stress were studied in number of plant species. For example, we have eight putative PAL genes in the genome of *Brachypodium* with *BdPAL1* and *BdPAL2* both playing a major role in the biogenesis of lignin and also both being expressed at a higher rate in stem tissues that form lignin as compared to the other six *BdPAL* genes. The knockdown of *BdPAL* lead to a surge in susceptibility to *M. oryzae* and *Fusarium culmorum* [63].

Interestingly, various genes involved in BSR have been discovered in crops [197], only a few have been utilized due to consequent yield penalty [35,198] and none of the BSR genes formerly identified, coding for proteins possessing RNA-binding activity by [108,199,200,201]. However, Zhou X. et al. [202] identified broad spectrum resistance Kitaake-1 (bsr-k1) mutant conferring BSR against *X. oryzae pvoryzae* and *Magnaporthe oryzae* with no consequent effect on yield in rice. It was revealed that the mRNAs of *OsPAL* genes which are associated with defense in plants was bounded by bsr-k1 protein, thus, promoting their turnover. Furthermore, the *OsPAL* mRNA build-up in the bsr-k1 mutant was a result of decline in mRNA turnover due to the loss of function of bsr-k.

## 8. Research Questions and Future Prospects

**Figure 4:**
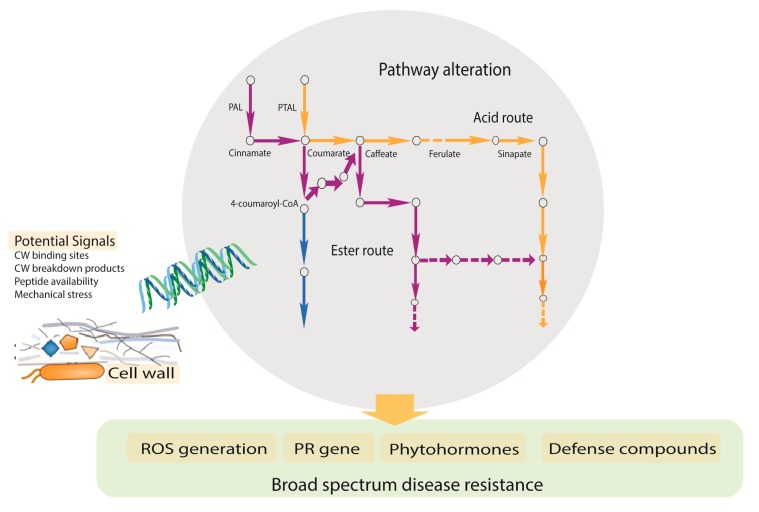
Schematic diagram demonstrating putative responses arising from the phenylpropanoid pathway towards plant disease resistance.

The phenylpropanoid pathway is an important center of attraction because of its importance in the production of defense related compounds and also because it has great involvement against pathogen attempts of cell wall weakening strategies. Advances in the last decade have supported that pathway engineering in plants can bring satisfactory solutions, keeping broad spectrum disease resistance and yield security in parallel. The plethora of available research on the phenylpropanoid pathway continue to show evidence of its importance and efficiency. In this review, we discussed all the possible aspects related to the phenylpropanoid pathway which could be important elements for disease resistance mechanisms (Figure 4) including involvement of transcriptome factors, metabolites derived from the pathway, novel gene families, and effects after engineering. Furthermore, we highlighted the emerging key players (PALs) in plant science from the phenylpropanoid pathway for concrete solutions for broad spectrum and yield penalties. In addition, from the previous investigations, we have noted some unsolved mysteries like (1) what is the importance of altering the lignin pathway for basal defense? (2) What are the candidate genes of the transcription factors, regulating multiple functions? (3) What are the key families from the phenylpropanoid pathway that putatively contribute to plant defense? The information available to us from the defense point of view is still fragmentary. It is elusive to the predict the function after alteration in phenylpropanoid pathway from the plant defense point of view. A deeper investigation is required to solve the mysteries raised above, which could provide valuable insights to aid engineering strategies for profitably tailoring plants.

## Figures and Tables

**Table 1:** Response of phenylpropanoid pathway genes against plant disease.

Gene Name	Crop	Pathogen Tested	Expression	Immune Response	References
**PAL**	*Linum* *uslerotiorum*	Multiple pathogens			[26]
	*Triticum aestivum*	Powdery mildew	Suppression	S	[60]
	*Nicotiana tabacum*	*Cercosporanicotianae*	Overexpression	R	[61]
	*Arabidopsis thaliana*	*Pseudomonas syringae*		S	[62,63]
	*Brachypodium*	*Magnaporthe* *Fusarium cuimorum*	Knockdown	S	[63]
	*Glycine max*	*Pseudomonas syringae*	Silencing	S	[64]
	*Capsicum annum*	*Xanthomonas*	Suppression	S	[65]
**C4H**	*Glycine max*	*Phytophthora sojae*	Overexpression	R	[66]
**4CL**	*Oryza sativa*	Rice blast		S	[67]
**HCT**	*Arabidopsis thaliana*	*Colletotrichum* *trifolli*			[68]
	*Medicago sativa*	Multiple pathogens			[69]
**CCR**	*Camelina sativa*	*Sclerotinia sclerotiorum*			[70]
**CCoAOMT**	*Triticum aestivum*	Powdery mildew			[60]
	*Zea mays*	Multiple pathogens		R	[71]
**COMT**	*Triticum aestivum*	*Rhizoctonia cerealis*	Silences	S	[72]
	*Triticum aestivum*	Powdery mildew			[60]
	*Arabidopsis* *thaliana*	*X. campestris, P. syringae,* *Hyaloperonospora*		R	[73]
		*Alternaria brassicicola* *B. cinerea, Blumeria graminis,*		S	[74]
	*Nicotiana tabacum*	*Agrobacterium tumefaciens*	Antisense	R	[75]
	*Sorghum bicolor*	*F. thapsinum*, *F. proliferatum*,		R	[76]
**CAD**	*Triticum aestivum*	Powdery mildew		S	[49]
	*Linum usitatissimum*	*F. oxysporum*	RNAi		[77]
	*Arabidopsis*	*Pseudomonas syringae*		S	[78]
	*Sorghum bicolor*	*Alternaria alternate* *F. verticillioides*, *F. proliferatum*, *Fusarium thapsinum*,		R	[76]
**F5H**	*Arabidopsis thaliana*	*Verticillium longisporum* *Sclerotinia sclerotiorum*		S S	[79,80]

Italics: R-enhanced resistance; S-enhanced susceptibility.

**Table 2:** Classes of transcription factors that regulate phenylpropanoid pathway biosynthetic genes potentially involved in defense.

Class	TFs	Gene Pathway/Enzyme Gene	Crop	Reference
**MYB**	*AtMYB4*	Sinapate esters	*Arabidopsis thaliana*	[93,98,99]
	*AtMYB32*	Reduction of lignin, CoMT	*Arabidopsis thaliana*	[100]
	*AtMYB15*	Lignification	*Arabidopsis thaliana*	[101]
	*AmMYB330*	Lignin and increased G/S ratio 4Cl, CAD	*Antirrhinum majus*	[96]
	*PvMYB4*	Reduced lignin, PAL CCoAOMT	*Panicum virgatum* L.	[102]
	*ZmMYB8, ZmMYB11, ZmMYB31, ZmMYB42*	Reduced lignin, COMT, PAL and 4CL	*Zea mays/Arabidopsis thaliana*	[103,104,105]
	*PtMYB14*	4CL	*Pinus taeda*	[106,107]
	*CsMYB4a*	Reduced lignin content And phenylalanine PAL, CCoAMT, 4CL, COMT	*Camellia sinensis/Nicotiana tabacum*	[108]
	*SmMYB39*	Reduced 4-coumaric acid, PAL. 4CL	*Salvia miltiorrhiza*	[109]
	*LlMYB1*	Reduced lignin PAL, 4CL	*Leucaena leucocephala*	[110]
	*TaMYB4*	Reduced lignin CCD, CCR	*Triticum aestivum* L.	[111]
	*AtMYB052/AtMYB054/ AtMYB069*	Cell wall thickening	*Arabidopsis thaliana*	[112]
	*EjMYB1*	Activate lignin biosynthetic genes	*Eriobotrya japonica*	[113]
	*PtoMYB216*	Lignin biosynthetic pathway	*Populus tomentosa*	[114]
	*PtoMYB156*	Repress phenylpropanoid biosynthesis	*Populus tomentosa*	[115]
**WRKY**	*PtrWRKY19*	Negatively regulate pith SCW	*Populus trichocarpa*	[116]
	*StWRKY8*	Phenylpropanoid metabolites	*Solanum tuberosum*	[117]
	*VvWRKY2*	Affect S/G ratio.	*Vitis vinifera* L	[118]
**HD-Zip**	*popREVOLUTA (PRE)*	Secondary vascular tissues	*Populus trichocarpa*	[119]
	POPCORONA (PCN)	SCW lignification	*Populus tremula* × *alba*	[120]
	*EcHB1*	Lignin and hemicellulose content	*Eucalyptus camaldulensis*	[121]
**SWN**	*PtrSND1-A2 (PtrWND1B)*	Cell wall thickening	*Populus trichocarpa*	[122,123]
	*PtoVNS11*	Regulate lignin deposition	*Populus tomentosa*	[124]
**ERF**	*PsnSHN2*	Negatively regulate lignin biosynthesis	*Populus simonii* × *nigra*	[67]

**Table 3:** Recent studies demonstrating the coumarin accumulation during plant-pathogen infection.

Plant	Plant Tissue	Disease	Coumarins	Reference
***Hevea brasiliensi***	Leaves	*Phytophthora palmivora*	Scopoletin	[146]
***Pisum sativum***	Leaves	*Uromyces pisi*	Scopoletin	[147]
***Brassica oleracea***	Leaves	*Xanthomonas campestris*	Coumarin	[148]
***Solanum lycopersicum***	Leaves	Tomato yellow leaf curl virus	Scopoletin	[149]
***Nicotiana tabacum***	Leaves, roots	*Botrytis cinereal*	Scopoletin, Esculin, Fraxetin	[150,151,152]
***Arabidopsis thaliana***	Leaves, roots	*Paeni bacilluspolymyxa* *Pseudomonas fluorescens* *Pythium sylvaticum*	Scopoletin, Esculin, Esculetin	[153] [100] [154]

## References

[1] Lionetti V., Métraux J.-P. (2014). Plant cell wall in pathogenesis, parasitism and symbiosis. Front. Plant Sci.

[2] Höfte H., Voxeur A. (2017). Plant cell walls. Curr. Biol..

[3] Hoson T., Wakabayashi K. (2015). Role of the plant cell wall in gravity resistance. Phytochemistry.

[4] Toth I.K., Birch P.R.J. (2005). Rotting softly and stealthily. Curr. Opin. Plant Biol..

[5] Bacete L., Mélida H., Miedes E., Molina A. (2018). Plant cell wall-mediated immunity: Cell wall changes trigger disease resistance responses. Plant J..

[6] Pogorelko G., Lionetti V., Bellincampi D., Zabotina O. (2013). Cell wall integrity. Plant Signal. Behav..

[7] De Lorenzo G., Ferrari S. (2002). Polygalacturonase-inhibiting proteins in defense against phytopathogenic fungi. Curr. Opin. Plant Biol..

[8] Vogel J.P., Raab T.K., Somerville C.R., Somerville S.C. (2004). Mutations in PMR5 result in powdery mildew resistance and altered cell wall composition. Plant J..

[9] Rui Y., Dinneny J.R. (2020). A wall with integrity: Surveillance and maintenance of the plant cell wall under stress. New Phytol..

[10] Engelsdorf T., Hamann T. (2014). An update on receptor-like kinase involvement in the maintenance of plant cell wall integrity. Ann. Bot..

[11] Wolf S. (2017). Plant cell wall signalling and receptor-like kinases. Biochem. J..

[12] Engelsdorf T., Gigli-Bisceglia N., Veerabagu M., McKenna J.F., Vaahtera L., Augstein F., Van der Does D., Zipfel C., Hamann T. (2018). The plant cell wall integrity maintenance and immune signaling systems cooperate to control stress responses in *Arabidopsis thaliana*. Sci. Signal..

[13] Boller T., Felix G. (2009). A Renaissance of elicitors: Perception of microbe-associated molecular patterns and danger signals by pattern-recognition receptors. Annu. Rev. Phytopathol..

[14] Simon C., Lion C., Biot C., Gierlinger N., Hawkins S. (2018). Lignification and Advances in Lignin Imaging in Plant Cell Walls. Annu. Plant Rev. Online.

[15] Denancé N., Ranocha P., Oria N., Barlet X., Rivière M.-P., Yadeta K.A., Hoffmann L., Perreau F., Clément G., Maia-Grondard A. (2013). Arabidopsis wat1 (walls are thin1)-mediated resistance to the bacterial vascular pathogen, Ralstonia solanacearum, is accompanied by cross-regulation of salicylic acid and tryptophan metabolism. Plant J..

[16] Jaeck E., Dumas B., Geoffroy P., Favet N., Inze D., Van Montagu M., Fritig B., Legrand M. (1992). Regulation of enzymes involved in lignin biosynthesis: Induction of O-methyltransferase mRNAs during the hypersensitive reaction of tobacco to tobacco mosaic virus. Mol. Plant Microbe Int..

[17] Chowdhury J., Henderson M., Schweizer P., Burton R.A., Fincher G.B., Little A. (2014). Differential accumulation of callose, arabinoxylan and cellulose in nonpenetrated versus penetrated papillae on leaves of barley infected with *Blumeria graminis* f. sp. *hordei*. New Phytol..

[18] Chowdhury J., Schober M.S., Shirley N.J., Singh R.R., Jacobs A.K., Douchkov D., Schweizer P., Fincher G.B., Burton R.A., Little A. (2016). Down-regulation of the *glucan synthase-like 6 gene (HvGsl6)* in barley leads to decreased callose accumulation and increased cell wall penetration by *Blumeria graminis* f. sp. *hordei*. New Phytol..

[19] Kwon C., Neu C., Pajonk S., Yun H.S., Lipka U., Humphry M., Bau S., Straus M., Kwaaitaal M., Rampelt H. (2008). Co-option of a default secretory pathway for plant immune responses. Nature.

[20] Vorwerk S., Somerville S., Somerville C. (2004). The role of plant cell wall polysaccharide composition in disease resistance. Trends Plant Sci..

[21] Wuyts N., Swennen R., Waele D.D. (2006). Effects of plant phenylpropanoid pathway products and selected terpenoids and alkaloids on the behaviour of the plant-parasitic nematodes Radopholus similis, Pratylenchus penetrans and Meloidogyne incognita. Nematology.

[22] Zhao R., Nakamura T., Fu Y., Lazar Z., Spector D.L. (2011). Gene bookmarking accelerates the kinetics of post-mitotic transcriptional re-activation. Nat. Cell Biol..

[23] Ahuja I., Kissen R., Bones A.M. (2012). Phytoalexins in defense against pathogens. Trends Plant Sci..

[24] Zhang S.-H., Yang Q., Ma R.-C. (2007). *Erwinia carotovora* ssp. carotovora Infection Induced “Defense Lignin” Accumulation and Lignin Biosynthetic Gene Expression in Chinese Cabbage (*Brassica rapa* L. ssp. *pekinensis*). J. Integr. Plant Biol..

[25] Eynck C., Koopmann B., Karlovsky P., von Tiedemann A. (2009). Internal Resistance in Winter Oilseed Rape Inhibits Systemic Spread of the Vascular Pathogen Verticillium longisporum. Phytopathology.

[26] Hano C., Martin I., Fliniaux O., Legrand B., Gutierrez L., Arroo R.R.J., Mesnard F., Lamblin F., Lainé E. (2006). Pinoresinol–lariciresinol reductase gene expression and secoisolariciresinol diglucoside accumulation in developing flax (Linum usitatissimum) seeds. Planta.

[27] Lorenzo O., Piqueras R., Sánchez-Serrano J.J., Solano R. (2003). ETHYLENE RESPONSE FACTOR1 Integrates Signals from Ethylene and Jasmonate Pathways in Plant Defense. Plant Cell.

[28] Anderson J.P., Badruzsaufari E., Schenk P.M., Manners J.M., Desmond O.J., Ehlert C., Maclean D.J., Ebert P.R., Kazan K. (2004). Antagonistic Interaction between Abscisic Acid and Jasmonate-Ethylene Signaling Pathways Modulates Defense Gene Expression and Disease Resistance in Arabidopsis. Plant Cell.

[29] Sattler S.E., Funnell-Harris D.L., Pedersen J.F. (2010). Brown midrib mutations and their importance to the utilization of maize, sorghum, and pearl millet lignocellulosic tissues. Plant Sci..

[30] Zhou J., Lee C., Zhong R., Ye Z.-H. (2009). MYB58 and MYB63 Are Transcriptional Activators of the Lignin Biosynthetic Pathway during Secondary Cell Wall Formation in Arabidopsis. Plant Cell.

[31] Zhong R., Ye Z.-H. (2009). Transcriptional regulation of lignin biosynthesis. Plant Signal. Behav..

[32] Scully E.D., Gries T., Sarath G., Palmer N.A., Baird L., Serapiglia M.J., Dien B.S., Boateng A.A., Ge Z., Funnell-Harris D.L. (2016). Overexpression of SbMyb60 impacts phenylpropanoid biosynthesis and alters secondary cell wall composition in sorghum bicolor. Plant J..

[33] Ma D., Constabel C.P. (2019). MYB repressors as regulators of phenylpropanoid metabolism in plants. Trends Plant Sci..

[34] Ning Y., Wang G.-L. (2018). Breeding plant broad-spectrum resistance without yield penalties. Proc. Natl. Acad. Sci. USA.

[35] Ning Y., Liu W., Wang G.-L. (2017). Balancing Immunity and Yield in Crop Plants. Trends Plant Sci..

[36] Bonawitz N.D., Chapple C. (2010). The Genetics of Lignin Biosynthesis: Connecting Genotype to Phenotype. Plant Physiol..

[37] Miedes E., Vanholme R., Boerjan W., Molina A. (2014). The role of the secondary cell wall in plant resistance to pathogens. Front. Plant Sci..

[38] Hao Z., Mohnen D. (2014). A review of xylan and lignin biosynthesis: Foundation for studying Arabidopsis irregular xylem mutants with pleiotropic phenotypes. Crit. Rev. Biochem. Mol. Biol..

[39] Barros J., Serk H., Granlund I., Pesquet E. (2015). The cell biology of lignification in higher plants. Ann. Bot..

[40] Vanholme R., De Meester B., Ralph J., Boerjan W. (2019). Lignin biosynthesis and its integration into metabolism. Curr. Opin. Biotechnol..

[41] Humphreys J.M., Chapple C. (2002). Rewriting the lignin roadmap. Curr. Opin. Plant Biol..

[42] Vanholme R., Cesarino I., Rataj K., Xiao Y., Sundin L., Goeminne G., Kim H., Cross J., Morreel K., Araujo P. (2013). Caffeoyl Shikimate Esterase (CSE) Is an Enzyme in the Lignin Biosynthetic Pathway in Arabidopsis. Science.

[43] Buendgen M.R., Coors J.G., Grombacher A.W., Russell W.A. (1990). European Corn Borer Resistance and Cell Wall Composition of Three Maize Populations. Crop Sci..

[44] Bonello P., Blodgett J.T. (2003). Pinus nigra–Sphaeropsis sapinea as a model pathosystem to investigate local and systemic effects of fungal infection of pines. Physiol. Mol. Plant Pathol..

[45] Dicko M.H., Gruppen H., Barro C., Traore A.S., van Berkel W.J.H., Voragen A.G.J. (2005). Impact of Phenolic Compounds and Related Enzymes in Sorghum Varieties for Resistance and Susceptibility to Biotic and Abiotic Stresses. J. Chem. Ecol..

[46] Lozovaya V.V., Lygin A.V., Zernova O.V., Ulanov A.V., Li S., Hartman G.L., Widholm J.M. (2007). Modification of phenolic metabolism in soybean hairy roots through down regulation of chalcone synthase or isoflavone synthase. Planta.

[47] Naoumkina M.A., He X., Dixon R.A. (2008). Elicitor-induced transcription factors for metabolic reprogramming of secondary metabolism in Medicago truncatula. BMC Plant Biol..

[48] Boerjan W., Ralph J., Baucher M. (2003). Lignin biosynthesis. Annu. Rev. Plant Biol..

[49] Bhuiyan N.H., Liu W., Liu G., Selvaraj G., Wei Y., King J. (2007). Transcriptional regulation of genes involved in the pathways of biosynthesis and supply of methyl units in response to powdery mildew attack and abiotic stresses in wheat. Plant Mol. Biol..

[50] Zhao J., Buchwaldt L., Rimmer S.R., Sha A., Mcgregor L., Bekkaoui D., Hegdues D. (2009). Patterns of differential gene expression in Brassica napus cultivars infected with Sclerotinia sclerotiorum. Mol. Plant. Pathol..

[51] Weng J.-K., Mo H., Chapple C. (2010). Over-expression of F5H in COMT-deficient Arabidopsis leads to enrichment of an unusual lignin and disruption of pollen wall formation. Plant J..

[52] Chen F., Tobimatsu Y., Havkin-Frenkel D., Dixon R.A., Ralph J. (2012). A polymer of caffeyl alcohol in plant seeds. Proc. Natl. Acad. Sci. USA.

[53] Yu O., Jung W., Shi J., Croes R.A., Fader G.M., McGonigle B., Odell J.T. (2000). Production of the Isoflavones Genistein and Daidzein in Non-Legume Dicot and Monocot Tissues. Plant Physiol..

[54] Yu O., Jez J.M. (2008). Nature’s assembly line: Biosynthesis of simple phenylpropanoids and polyketides. Plant J..

[55] Dixon R.A., Achnine L., Kota P., Liu C.-J., Reddy M.S.S., Wang L. (2002). The phenylpropanoid pathway and plant defence—a genomics perspective. Mol. Plant Pathol..

[56] Dixon L.J., Castlebury L.A., Aime M.C., Glynn N.C., Comstock J.C. (2010). Phylogenetic relationships of sugarcane rust fungi. Mycol. Prog..

[57] Gallego-Giraldo L., Posé S., Pattathil S., Peralta A.G., Hahn M.G., Ayre B.G., Sunuwar J., Hernandez J., Patel M., Shah J. (2018). Elicitors and defense gene induction in plants with altered lignin compositions. New Phytol..

[58] Le Roy J., Huss B., Creach A., Hawkins S., Neutelings G. (2016). Glycosylation Is a Major Regulator of Phenylpropanoid Availability and Biological Activity in Plants. Front. Plant Sci..

[59] Zhao Q., Dixon R.A. (2014). Altering the Cell Wall and Its Impact on Plant Disease: From Forage to Bioenergy. Annu. Rev. Phytopathol..

[60] Bhuiyan N.H., Selvaraj G., Wei Y., King J. (2009). Gene expression profiling and silencing reveal that monolignol biosynthesis plays a critical role in penetration defence in wheat against powdery mildew invasion. J. Exp. Bot..

[61] Shadle G.L., Wesley S.V., Korth K.L., Chen F., Lamb C., Dixon R.A. (2003). Phenylpropanoid compounds and disease resistance in transgenic tobacco with altered expression of l-phenylalanine ammonia-lyase. Phytochemistry.

[62] Huang J., Gu M., Lai Z., Fan B., Shi K., Zhou Y.-H., Yu J.-Q., Chen Z. (2010). Functional Analysis of the Arabidopsis PAL Gene Family in Plant Growth, Development, and Response to Environmental Stress. Plant Physiol..

[63] Cass C.L., Peraldi A., Dowd P.F., Mottiar Y., Santoro N., Karlen S.D., Bukhman Y.V., Foster C.E., Thrower N., Bruno L.C. (2015). Effects of phenylalanine ammonia lyase (PAL) knockdown on cell wall composition, biomass digestibility, and biotic and abiotic stress responses in Brachypodium. J. Exp. Bot..

[64] Shine M.B., Yang J.-W., El-Habbak M., Nagyabhyru P., Fu D.-Q., Navarre D., Ghabrial S., Kachroo P., Kachroo A. (2016). Cooperative functioning between phenylalanine ammonia lyase and isochorismate synthase activities contributes to salicylic acid biosynthesis in soybean. New Phytol..

[65] Kim D.S., Hwang B.K. (2014). An important role of the pepper phenylalanine ammonia-lyase gene (PAL1) in salicylic acid-dependent signalling of the defence response to microbial pathogens. J. Exp. Bot..

[66] Yan Q., Si J., Cui X., Peng H., Chen X., Xing H., Dou D. (2019). The soybean cinnamate 4-hydroxylase gene GmC4H1 contributes positively to plant defense via increasing lignin content. Plant Growth Regul..

[67] Liu Y., Wei M., Hou C., Lu T., Liu L., Wei H., Cheng Y., Wei Z. (2017). Functional characterization of populus psnshn2 in coordinated regulation of secondary wall components in tobacco. Sci. Rep..

[68] Gallego-Giraldo L., Escamilla-Trevino L., Jackson L.A., Dixon R.A. (2011). Salicylic acid mediates the reduced growth of lignin down-regulated plants. Proc. Natl. Acad. Sci. USA.

[69] Gallego-Giraldo L., Jikumaru Y., Kamiya Y., Tang Y., Dixon R.A. (2011). Selective lignin downregulation leads to constitutive defense response expression in alfalfa (*Medicago sativa* L.). New Phytol..

[70] Eynck C., Seguin-Swartz G., Clarke W.E., Parkin I.A.P. (2012). Monolignol biosynthesis is associated with resistance to Sclerotinia sclerotiorum in Camelina sativa. Mol. Plant Pathol..

[71] Yang Q., He Y., Kabahuma M., Chaya T., Kelly A., Borrego E., Bian Y., El Kasmi F., Yang L., Teixeira P. (2017). A gene encoding maize caffeoyl-CoA O-methyltransferase confers quantitative resistance to multiple pathogens. Nat. Genet..

[72] Wang M., Zhu X., Wang K., Lu C., Luo M., Shan T., Zhang Z. (2018). A wheat caffeic acid 3-O-methyltransferase TaCOMT-3D positively contributes to both resistance to sharp eyespot disease and stem mechanical strength. Sci. Rep..

[73] Quentin M., Allasia V., Pegard A., Allais F., Ducrot P.-H., Favery B., Levis C., Martinet S., Masur C., Ponchet M. (2009). Imbalanced Lignin Biosynthesis Promotes the Sexual Reproduction of Homothallic Oomycete Pathogens. PLOS Pathog..

[74] Aharoni A., Dixit S., Jetter R., Thoenes E., van Arkel G., Pereira A. (2004). The SHINE clade of AP2 domain transcription factors activates wax biosynthesis, alters cuticle properties, and confers drought tolerance when overexpressed in Arabidopsis. Plant Cell.

[75] Maury S., Delaunay A., Mesnard F., Crônier D., Chabbert B., Geoffroy P., Legrand M. (2010). O-methyltransferase(s)-suppressed plants produce lower amounts of phenolic vir inducers and are less susceptible to Agrobacterium tumefaciens infection. Planta.

[76] Funnell-Harris D.L., Pedersen J.F., Sattler S.E. (2010). Alteration in Lignin Biosynthesis Restricts Growth of Fusarium spp. in Brown Midrib Sorghum. Phytopathology.

[77] Wróbel-Kwiatkowska M., Starzycki M., Zebrowski J., Oszmiański J., Szopa J. (2007). Lignin deficiency in transgenic flax resulted in plants with improved mechanical properties. J. Biotechnol..

[78] Sibout R., Eudes A., Mouille G. (2005). Cinnamyl alcohol dehydrogenase-C and -D are the primary genes involved in lignin biosynthesis in the floral stem of Arabidopsis. Plant Cell.

[79] Huang J., Bhinu V.S., Li X., Dallal Bashi Z., Zhou R., Hannoufa A. (2009). Pleiotropic changes in Arabidopsis f5h and sct mutants revealed by large-scale gene expression and metabolite analysis. Planta.

[80] König S., Feussner K., Kaever A., Landesfeind M., Thurow C., Karlovsky P., Gatz C., Polle A., Feussner I. (2014). Soluble phenylpropanoids are involved in the defense response of Arabidopsis against Verticillium longisporum. New Phytol..

[81] Zhao Q., Dixon R.A. (2011). Transcriptional networks for lignin biosynthesis: More complex than we thought?. Trends Plant Sci..

[82] Gachon C.M.M., Langlois-Meurinne M., Henry Y., Saindrenan P. (2005). Transcriptional co-regulation of secondary metabolism enzymes in Arabidopsis: Functional and evolutionary implications. Plant Mol. Biol..

[83] Naoumkina M.A., Zhao Q., Gallego-Giraldo L., Dai X., Zhao P.X., Dixon R.A. (2010). Genome-wide analysis of phenylpropanoid defence pathways. Mol. Plant Pathol..

[84] Rao X., Dixon R.A. (2018). Current Models for Transcriptional Regulation of Secondary Cell Wall Biosynthesis in Grasses. Front. Plant Sci..

[85] Ohtani M., Demura T. (2019). The quest for transcriptional hubs of lignin biosynthesis: Beyond the NAC-MYB-gene regulatory network model. Curr. Opin. Biotechnol..

[86] Lacombe E., Van Doorsselaere J., Boerjan W., Boudet A.M., Grima-Pettenati J. (2000). Characterization of cis-elements required for vascular expression of the Cinnamoyl CoA Reductase gene and for protein–DNA complex formation. Plant J..

[87] Legay S., Lacombe E., Goicoechea M., Brière C., Séguin A., Mackay J., Grima-Pettenati J. (2007). Molecular characterization of EgMYB1, a putative transcriptional repressor of the lignin biosynthetic pathway. Plant Sci..

[88] Mitsuda N., Iwase A., Yamamoto H., Yoshida M., Seki M., Shinozaki K., Ohme-Takagi M. (2007). NAC Transcription Factors, NST1 and NST3, Are Key Regulators of the Formation of Secondary Walls in Woody Tissues of Arabidopsis. Plant Cell.

[89] Zhao Q., Gallego-Giraldo L., Wang H., Zeng Y., Ding S.-Y., Chen F., Dixon R.A. (2010). An NAC transcription factor orchestrates multiple features of cell wall development in Medicago truncatula. Plant J..

[90] McCarthy R.L., Zhong R., Ye Z.-H. (2009). MYB83 Is a Direct Target of SND1 and Acts Redundantly with MYB46 in the Regulation of Secondary Cell Wall Biosynthesis in Arabidopsis. Plant Cell Physiol..

[91] Zhong R., Richardson E.A., Ye Z.-H. (2007). The MYB46 Transcription Factor Is a Direct Target of SND1 and Regulates Secondary Wall Biosynthesis in Arabidopsis. Plant Cell.

[92] Patzlaff A., McInnis S., Courtenay A., Surman C., Newman L.J., Smith C., Bevan M.W., Mansfield S., Whetten R.W., Sederoff R.R. (2003). Characterisation of a pine MYB that regulates lignification. Plant J..

[93] Jin H., Cominelli E., Bailey P., Parr A., Mehrtens F., Jones J., Tonelli C., Weisshaar B., Martin C. (2000). Transcriptional repression by AtMYB4 controls production of UV-protecting sunscreens in Arabidopsis. EMBO J..

[94] Mele G., Ori N., Sato Y., Hake S. (2003). The knotted1-like homeobox gene BREVIPEDICELLUS regulates cell differentiation by modulating metabolic pathways. Genes Dev..

[95] Sonbol F.-M., Fornalé S., Capellades M., Encina A., Touriño S., Torres J.-L., Rovira P., Ruel K., Puigdomènech P., Rigau J. (2009). The maize ZmMYB42 represses the phenylpropanoid pathway and affects the cell wall structure, composition and degradability in *Arabidopsis thaliana*. Plant Mol. Biol..

[96] Tamagnone L., Merida A., Parr A., Mackay S., Culianez-Macia F.A., Roberts K., Martin C. (1998). The AmMYB308 and AmMYB330 Transcription Factors from Antirrhinum Regulate Phenylpropanoid and Lignin Biosynthesis in Transgenic Tobacco. Plant Cell.

[97] Liang Y.-K., Dubos C., Dodd I.C., Holroyd G.H., Hetherington A.M., Campbell M.M. (2005). AtMYB61, an R2R3-MYB Transcription Factor Controlling Stomatal Aperture in *Arabidopsis thaliana*. Curr. Biol..

[98] Zhao J., Zhang W., Zhao Y., Gong X., Guo L., Zhu G., Wang X., Gong Z., Schumaker K.S., Guo Y. (2007). SAD2, an Importin β-Like Protein, Is Required for UV-B Response in arabidopsis by mediating MYB4 nuclear trafficking. Plant Cell.

[99] Zhou M., Sun Z., Wang C., Zhang X., Tang Y., Zhu X., Shao J., Wu Y. (2015). Changing a conserved amino acid in R2R3-MYB transcription repressors results in cytoplasmic accumulation and abolishes their repressive activity in Arabidopsis. Plant Cell.

[100] Preston J., Wheeler J., Heazlewood J., Li S.F., Parish R.W. (2004). AtMYB32 is required for normal pollen development in *Arabidopsis thaliana*. Plant Cell.

[101] Chezem W.R., Memon A., Li F.-S., Weng J.-K., Clay N.K. (2017). SG2-Type R2R3-MYB Transcription Factor MYB15 Controls Defense-Induced Lignification and Basal Immunity in Arabidopsis. Plant Cell.

[102] Shen H., He X., Poovaiah C.R., Wuddineh W.A., Ma J., Mann D.G.J., Wang H., Jackson L., Tang Y., Neal Stewart C. (2012). Functional characterization of the switchgrass (Panicum virgatum) R2R3-MYB transcription factor PvMYB4 for improvement of lignocellulosic feedstocks. New Phytol..

[103] Fornalé S., Shi X., Chai C., Encina A., Irar S., Capellades M., Fuguet E., Torres J.-L., Rovira P., Puigdomènech P. (2010). ZmMYB31 directly represses maize lignin genes and redirects the phenylpropanoid metabolic flux. Plant J..

[104] Vélez-Bermúdez I.-C., Salazar-Henao J.E., Fornalé S., López-Vidriero I., Franco-Zorrilla J.-M., Grotewold E., Gray J., Solano R., Schmidt W., Pagés M. (2015). A MYB/ZML Complex Regulates Wound-Induced Lignin Genes in Maize. Plant Cell.

[105] Fornalé S., Sonbol F.-M., Maes T., Capellades M., Puigdomènech P., Rigau J., Caparrós-Ruiz D. (2006). Down-regulation of the maize and *Arabidopsis thaliana* caffeic acid O-methyl-transferase genes by two new maize R2R3-MYB transcription factors. Plant Mol. Biol..

[106] Bedon F., Bomal C., Caron S., Levasseur C., Boyle B., Mansfield S.D., Schmidt A., Gershenzon J., Grima-Pettenati J., Séguin A. (2010). Subgroup 4 R2R3-MYBs in conifer trees: Gene family expansion and contribution to the isoprenoid- and flavonoid-oriented responses. J. Exp. Bot..

[107] Bomal C., Duval I., Giguère I., Fortin É., Caron S., Stewart D., Boyle B., Séguin A., MacKay J.J. (2013). Opposite action of R2R3-MYBs from different subgroups on key genes of the shikimate and monolignol pathways in spruce. J. Exp. Bot..

[108] Li M., Yasuda M., Yamaya-Ito H., Maeda M., Sasaki N., Nagata M., Suzuki A., Okazaki S., Sekimoto H., Yamada T. (2017). Involvement of programmed cell death in suppression of the number of root nodules formed in soybean induced by Bradyrhizobium infection. Soil Sci. Plant Nutr..

[109] Liu J., Ding P., Sun T., Nitta Y., Dong O., Huang X., Yang W., Li X., Botella J.R., Zhang Y. (2013). Heterotrimeric G Proteins Serve as a Converging Point in Plant Defense Signaling Activated by Multiple Receptor-Like Kinases. Plant Physiol..

[110] Omer S., Kumar S., Khan B.M. (2013). Over-expression of a subgroup 4 R2R3 type MYB transcription factor gene from Leucaena leucocephala reduces lignin content in transgenic tobacco. Plant Cell Rep..

[111] Ma Q.-H., Wang C., Zhu H.-H. (2011). TaMYB4 cloned from wheat regulates lignin biosynthesis through negatively controlling the transcripts of both cinnamyl alcohol dehydrogenase and cinnamoyl-CoA reductase genes. Biochimie.

[112] Zhong R., Lee C., Zhou J., McCarthy R.L., Ye Z.-H. (2008). A Battery of Transcription Factors Involved in the Regulation of Secondary Cell Wall Biosynthesis in Arabidopsis. Plant Cell.

[113] Xu Q., Yin X.-r., Zeng J.-k., Ge H., Song M., Xu C.-j., Li X., Ferguson I.B., Chen K.-s. (2014). Activator- and repressor-type MYB transcription factors are involved in chilling injury induced flesh lignification in loquat via their interactions with the phenylpropanoid pathway. J. Exp. Bot..

[114] Tian Q., Wang X., Li C., Lu W., Yang L., Jiang Y., Luo K. (2013). Functional characterization of the poplar R2R3-MYB transcription factor PtoMYB216 involved in the regulation of lignin biosynthesis during wood formation. PLoS ONE.

[115] Yang L., Zhao X., Ran L., Li C., Fan D., Luo K. (2017). PtoMYB156 is involved in negative regulation of phenylpropanoid metabolism and secondary cell wall biosynthesis during wood formation in poplar. Sci. Rep..

[116] Yang L., Zhao X., Yang F., Fan D., Jiang Y., Luo K. (2016). PtrWRKY19, a novel WRKY transcription factor, contributes to the regulation of pith secondary wall formation in Populus trichocarpa. Sci. Rep..

[117] Yogendra K.N., Kumar A., Sarkar K., Li Y., Pushpa D., Mosa K.A., Duggavathi R., Kushalappa A.C. (2015). Transcription factor StWRKY1 regulates phenylpropanoid metabolites conferring late blight resistance in potato. J. Exp. Bot..

[118] Guillaumie S., Mzid R., Méchin V., Léon C., Hichri I., Destrac-Irvine A., Trossat-Magnin C., Delrot S., Lauvergeat V. (2009). The grapevine transcription factor WRKY2 influences the lignin pathway and xylem development in tobacco. Plant Mol. Biol..

[119] Robischon M., Du J., Miura E., Groover A. (2011). The Populus Class III HD ZIP, popREVOLUTA, Influences Cambium Initiation and Patterning of Woody Stems. Plant Physiol..

[120] Du J., Miura E., Robischon M., Martinez C., Groover A. (2011). The Populus Class III HD ZIP Transcription Factor POPCORONA Affects Cell Differentiation during Secondary Growth of Woody Stems. PLoS ONE.

[121] Sonoda T., Koita H., Nakamoto-Ohta S., Kondo K., Suezaki T., Kato T., Kato T., Nagai K., Iida N., Sato S. (2009). Increasing fiber length and growth in transgenic tobacco plants overexpressing a gene encoding the Eucalyptus camaldulensis HD-Zip class II transcription factor. Plant Biotechnol..

[122] Li Q., Lin Y.-C., Sun Y.-H., Song J., Chen H., Zhang X.-H., Sederoff R.R., Chiang V.L. (2012). Splice variant of the SND1 transcription factor is a dominant negative of SND1 members and their regulation in Populus trichocarpa. Proc. Natl. Acad. Sci. USA.

[123] Zhao Y., Sun J., Xu P., Zhang R., Li L. (2014). Intron-mediated alternative splicing of wood-associated nac transcription factor1b regulates cell wall thickening during fiber development in populus species. Plant Physiol..

[124] Yang L., Hou Y., Zhao X., Lu W., Li Y., Yang F., Tang S., Luo K. (2015). Identification and characterization of a wood-associated NAC domain transcription factor PtoVNS11 from Populus tomentosa Carr. Trees.

[125] Nuhse T. (2012). Cell wall integrity signaling and innate immunity in plants. Front. Plant Sci..

[126] Donaldson L.A. (2001). Lignification and lignin topochemistry—an ultrastructural view. Phytochemistry.

[127] Bechinger C., Giebel K.-F., Schnell M., Leiderer P., Deising H.B., Bastmeyer M. (1999). Optical measurements of invasive forces exerted by appressoria of a plant pathogenic fungus. Science.

[128] Zhang Y., Wang X., Rong W., Yang J., Li Z., Wu L., Zhang G., Ma Z. (2017). Histochemical analyses reveal that stronger intrinsic defenses in gossypium barbadense than in g. hirsutum are associated with resistance to verticillium dahliae. Front. Plant Sci..

[129] Liu Q., Luo L., Zheng L. (2018). Lignins: Biosynthesis and biological functions in plants. Int. J. Mol. Sci..

[130] Voigt C.A. (2016). Cellulose/callose glucan networks: The key to powdery mildew resistance in plants?. New Phytol..

[131] Khan A., Li R.-J., Sun J.-T., Ma F., Zhang H.-X., Jin J.-H., Ali M., Haq S.u., Wang J.-E., Gong Z.-H. (2018). Genome-wide analysis of dirigent gene family in pepper (*Capsicum annuum* L.) and characterization of CaDIR7 in biotic and abiotic stresses. Sci. Rep..

[132] Santiago R., Barros-Rios J., Malvar R.A. (2013). Impact of cell wall composition on maize resistance to pests and diseases. Int. J. Mol. Sci..

[133] Mandal S., Kar I., Mukherjee A.K., Acharya P. (2013). Elicitor-induced defense responses in Solanum lycopersicum against Ralstonia solanacearum. Sci. World J..

[134] Tronchet M., Balague C., Kroj T., Jouanin L., Roby D. (2010). Cinnamyl alcohol dehydrogenases-C and D, key enzymes in lignin biosynthesis, play an essential role in disease resistance in Arabidopsis. Mol. Plant Pathol..

[135] Shi H., Liu Z., Zhu L., Zhang C., Chen Y., Zhou Y., Li F., Li X. (2012). Overexpression of cotton (Gossypium hirsutum) dirigent1 gene enhances lignification that blocks the spread of Verticillium dahliae. Acta Biochim. Biophys. Sin..

[136] Wang G.-F., He Y., Strauch R., Olukolu B.A., Nielsen D., Li X., Balint-Kurti P.J. (2015). Maize homologs of hydroxycinnamoyltransferase, a key enzyme in lignin biosynthesis, bind the nucleotide binding leucine-rich repeat rp1 proteins to modulate the defense response. Plant Physiol..

[137] Liu H., Guo Z., Gu F., Ke S., Sun D., Dong S., Liu W., Huang M., Xiao W., Yang G. (2017). 4-Coumarate-CoA Ligase-Like Gene OsAAE3 Negatively Mediates the Rice Blast Resistance, Floret Development and Lignin Biosynthesis. Front. Plant Sci..

[138] Bellincampi D., Cervone F., Lionetti V. (2014). Plant cell wall dynamics and wall-related susceptibility in plant–pathogen interactions. Front. Plant Sci..

[139] Zaynab M., Fatima M., Abbas S., Sharif Y., Umair M., Zafar M.H., Bahadar K. (2018). Role of secondary metabolites in plant defense against pathogens. Microb. Pathog..

[140] Stringlis I.A., de Jonge R., Pieterse C.M.J. (2019). The age of coumarins in plant–microbe interactions. Plant Cell Physiol..

[141] Kai K., Shimizu B.-i., Mizutani M., Watanabe K., Sakata K. (2006). Accumulation of coumarins in *Arabidopsis thaliana*. Phytochemistry.

[142] Gachon C., Baltz R., Saindrenan P. (2004). Over-expression of a scopoletin glucosyltransferase in Nicotiana tabacum leads to precocious lesion formation during the hypersensitive response to tobacco mosaic virus but does not affect virus resistance. Plant Mol. Biol..

[143] Shimizu B., Miyagawa H., Ueno T., Sakata K., Watanabe K., Ogawa K. (2014). Morning Glory Systemically Accumulates Scopoletin and Scopolin after Interaction with Fusarium oxysporum. Z. Für Naturforschung C.

[144] Chong J., Baltz R., Schmitt C., Beffa R., Fritig B., Saindrenan P. (2002). Downregulation of a pathogen-responsive tobacco udp-glc:phenylpropanoid glucosyltransferase reduces scopoletin glucoside accumulation, enhances oxidative stress, and weakens virus resistance. Plant Cell.

[145] Tanguy J., Martin C. (1972). Phenolic compounds and the hypersensitivity reaction in nicotiana tabacum infected with tobacco mosaic virus. Phytochemistry.

[146] Dutsadee C., Nunta C. (2008). Induction of peroxidase, scopoletin, phenolic compounds and resistance in Hevea brasiliensis by elicitin and a novel protein elicitor purified from phytophthora palmivora. Physiol. Mol. Plant Pathol..

[147] Barilli E., Rubiales D., Amalfitano C., Evidente A., Prats E. (2015). BTH and BABA induce resistance in pea against rust (Uromyces pisi) involving differential phytoalexin accumulation. Planta.

[148] Tortosa M., Cartea M.E., Rodríguez V.M., Velasco P. (2018). Unraveling the metabolic response of Brassica oleracea exposed to Xanthomonas campestris pv. campestris. J. Sci. of Food Agric..

[149] Sade D., Shriki O., Cuadros-Inostroza A., Tohge T., Semel Y., Haviv Y., Willmitzer L., Fernie A.R., Czosnek H., Brotman Y. (2015). Comparative metabolomics and transcriptomics of plant response to Tomato yellow leaf curl virus infection in resistant and susceptible tomato cultivars. Metabolomics.

[150] El Oirdi M., Trapani A., Bouarab K. (2010). The nature of tobacco resistance against Botrytis cinerea depends on the infection structures of the pathogen. Environ. Microbiol..

[151] Sun H., Wang L., Zhang B., Ma J., Hettenhausen C., Cao G., Sun G., Wu J., Wu J. (2014). Scopoletin is a phytoalexin against Alternaria alternata in wild tobacco dependent on jasmonate signalling. J. Exp. Bot..

[152] Santhanam R., Menezes R.C., Grabe V., Li D., Baldwin I.T., Groten K. (2019). A suite of complementary biocontrol traits allows a native consortium of root-associated bacteria to protect their host plant from a fungal sudden-wilt disease. Mol. Ecol..

[153] Chaouch S., Queval G., Noctor G. (2012). AtRbohF is a crucial modulator of defence-associated metabolism and a key actor in the interplay between intracellular oxidative stress and pathogenesis responses in Arabidopsis. Plant J..

[154] Stringlis I.A., Zhang H., Pieterse C.M.J., Bolton M.D., de Jonge R. (2018). Microbial small molecules—weapons of plant subversion. Nat. Prod. Rep..

[155] Treutter D. (2005). Significance of flavonoids in plant resistance and enhancement of their biosynthesis. Environ. Chem. Lett..

[156] Cushnie T.P.T., Lamb A.J. (2005). Antimicrobial activity of flavonoids. Int. J. Antimicrob. Agents.

[157] Tsuchiya H., Iinuma M. (2000). Reduction of membrane fluidity by antibacterial sophoraflavanone G isolated from sophora exigua. Phytomedicine.

[158] Tamba Y., Ohba S., Kubota M., Yoshioka H., Yoshioka H., Yamazaki M. (2007). Single GUV method reveals interaction of tea catechin (−)-epigallocatechin gallate with lipid membranes. Biophys. J..

[159] Katsumata S., Hamana K., Horie K., Toshima H., Hasegawa M. (2017). Identification of sternbin and naringenin as detoxified metabolites from the rice flavanone phytoalexin sakuranetin by pyricularia oryzae. Chem. Biodivers..

[160] Delaney T.P., Uknes S., Vernooij B., Friedrich L., Weymann K., Negrotto D., Gaffney T., Gut-Rella M., Kessmann H., Ward E. (1994). A central role of salicylic acid in plant disease resistance. Science.

[161] Lu H. (2009). Dissection of salicylic acid-mediated defense signaling networks. Plant Signal. Behav..

[162] Robert-Seilaniantz A., Grant M., Jones J.D.G. (2011). Hormone crosstalk in plant disease and defense: More than just jasmonate-salicylate antagonism. Annu. Rev. Phytopathol..

[163] Yuan W., Jiang T., Du K., Chen H., Cao Y., Xie J., Li M., Carr J.P., Wu B., Fan Z. (2019). Maize phenylalanine ammonia-lyases contribute to resistance to Sugarcane mosaic virus infection, most likely through positive regulation of salicylic acid accumulation. Mol. Plant Pathol..

[164] Zhang C., Wang X., Zhang F., Dong L., Wu J., Cheng Q., Qi D., Yan X., Jiang L., Fan S. (2017). Phenylalanine ammonia-lyase2.1 contributes to the soybean response towards Phytophthora sojae infection. Sci. Rep..

[165] Peng J., Deng X., Jia S., Huang J., Miao X., Huang Y. (2004). Role of Salicylic Acid in Tomato Defense against Cotton Bollworm, Helicoverpa armigera Hubner. Z. Für Naturforschung C.

[166] Mauch-Mani B., Slusarenko A.J. (1996). Production of salicylic acid precursors is a major function of phenylalanine ammonia-lyase in the resistance of arabidopsis to peronospora parasitica. Plant Cell.

[167] Duan C., Yu J., Bai J., Zhu Z., Wang X. (2014). Induced defense responses in rice plants against small brown planthopper infestation. Crop J..

[168] Kawasaki T., Koita H., Nakatsubo T., Hasegawa K., Wakabayashi K., Takahashi H., Umemura K., Umezawa T., Shimamoto K. (2006). Cinnamoyl-CoA reductase, a key enzyme in lignin biosynthesis, is an effector of small GTPase Rac in defense signaling in rice. Proc. Natl. Acad. Sci. USA.

[169] Hibino T., Takabe K., Kawazu T. (1995). Increase of Cinnamaldehyde Groups in Lignin of Transgenic Tobacco Plants Carrying an Antisense Gene for Cinnamyl Alcohol Dehydrogenase. Biosci Biotech Biochem.

[170] Chen L.-Q., Hou B.-H., Lalonde S., Takanaga H., Hartung M.L., Qu X.-Q., Guo W.-J., Kim J.-G., Underwood W., Chaudhuri B. (2010). Sugar transporters for intercellular exchange and nutrition of pathogens. Nature.

[171] Anil K., Das S.N., Podile A.R. (2014). Induced defense in plants: A short overview. Proc. Natl. Acad. Sci. USA India Sect. B Biol. Sci..

[172] Abdul Malik N.A., Kumar I., Nadarajah S. (2020). Orchestrators of plant defense and immunity. Int. J. Mol. Sci..

[173] Boureau T., Siamer S., Perino C., Gaubert S., Patrit O., Degrave A., Fagard M., Chevreau E., Barny M.-A. (2011). The HrpN effector of erwinia amylovora, Which is involved in Type III translocation, contributes directly or indirectly to callose elicitation on apple leaves. Mol. Plant Microbe Inter..

[174] Boureau T., ElMaarouf-Bouteau H., Garnier A., Brisset M.-N., Perino C., Pucheu I., Barny M.-A. (2006). DspA/E, a type iii effector essential for erwinia amylovora pathogenicity and growth in planta, induces cell death in host apple and nonhost tobacco plants. Mol. Plant Microbe Inter..

[175] Facchini P.J., Hagel J., Zulak K.G. (2002). Hydroxycinnamic acid amide metabolism: Physiology and biochemistry. Can. J. Bot..

[176] Vogt T. (2010). Phenylpropanoid biosynthesis. Mol. Plant.

[177] Thilmony R., Underwood W., He S.Y. (2006). Genome-wide transcriptional analysis of the *Arabidopsis thaliana* interaction with the plant pathogen pseudomonas syringae pv. tomato DC3000 and the human pathogen escherichia coli O157:H7. Plant J..

[178] Djamei A., Kahmann R. (2012). Ustilago maydis: Dissecting the molecular interface between pathogen and plant. PLOS Pathog..

[179] Jacobs J.M., Milling A., Mitra R.M., Hogan C.S., Ailloud F., Prior P., Allen C. (2013). Ralstonia solanacearum requires pops, an ancient avre-family effector, for virulence and to overcome salicylic acid-mediated defenses during tomato pathogenesis. mBio.

[180] Jelenska J., Yao N., Vinatzer B.A., Wright C.M., Brodsky J.L., Greenberg J.T. (2007). AJ domain virulence effector of Pseudomonas syringae remodels host chloroplasts and suppresses defenses. Curr. Biol. CB.

[181] Zhou H., Lin J., Johnson A., Morgan R.L., Zhong W., Ma W. (2011). Pseudomonas syringae type III effector hopz1 targets a host enzyme to suppress isoflavone biosynthesis and promote infection in soybean. Cell Host Microbe.

[182] Venisse J.-S., Malnoy M., Faize M., Paulin J.-P., Brisset M.-N. (2002). Modulation of Defense Responses of Malus spp. During Compatible and Incompatible Interactions with Erwinia amylovora. Mol. Plant Microbe Inter..

[183] Degrave A., Siamer S., Boureau T., Barny M.-A. (2015). The AvrE superfamily: Ancestral type III effectors involved in suppression of pathogen-associated molecular pattern-triggered immunity. Mol. Plant Pathol..

[184] Asselin J.A.E., Lin J., Perez-Quintero A.L., Gentzel I., Majerczak D., Opiyo S.O., Zhao W., Paek S.-M., Kim M.G., Coplin D.L. (2015). Perturbation of maize phenylpropanoid metabolism by an AvrE family type III effector from *Pantoea stewartii*. Plant Physiol..

[185] Oerke E.C. (2006). Crop losses to pests. J. Agric. Sci..

[186] An C., Mou Z. (2011). Salicylic Acid and its Function in Plant ImmunityF. J. Integr. Plant Biol..

[187] Fu D., Chen L., Yu G., Liu Y., Lou Q., Mei H., Xiong L., Li M., Xu X., Luo L. (2011). QTL mapping of sheath blight resistance in a deep-water rice cultivar. Euphytica.

[188] Zhou Y.-L., Xie X.-W., Xu M.-R., Zang J.-P., Zhu L.-H., Xu J.-L., Li Z.-K. (2012). Genetic Overlap in the Quantitative Resistance of Rice at the Seedling and Adult Stages to Xanthomonas oryzae pv. oryzae. J. Plant Biol..

[189] Fukuoka S., Okuno K. (2001). QTL analysis and mapping of pi21, a recessive gene for field resistance to rice blast in Japanese upland rice. Theor. Appl. Genet..

[190] Tonnessen B.W., Manosalva P., Lang J.M., Baraoidan M., Bordeos A., Mauleon R., Oard J., Hulbert S., Leung H., Leach J.E. (2015). Rice phenylalanine ammonia-lyase gene OsPAL4 is associated with broad spectrum disease resistance. Plant Mol. Biol..

[191] Liu H., Li X., Xiao J., Wang S. (2012). A convenient method for simultaneous quantification of multiple phytohormones and metabolites: Application in study of rice-bacterium interaction. Plant Methods.

[192] Shen X., Yuan B., Liu H., Li X., Xu C., Wang S. (2010). Opposite functions of a rice mitogen-activated protein kinase during the process of resistance against Xanthomonas oryzae. Plant J..

[193] Tao Z., Liu H., Qiu D., Zhou Y., Li X., Xu C., Wang S. (2009). A pair of allelic wrky genes play opposite roles in rice-bacteria interactions. Plant Physiol..

[194] Chuma I., Isobe C., Hotta Y., Ibaragi K., Futamata N., Kusaba M., Yoshida K., Terauchi R., Fujita Y., Nakayashiki H. (2011). Multiple translocation of the avr-pita effector gene among chromosomes of the rice blast fungus magnaporthe oryzae and related species. PLOS Pathog..

[195] Yuan B., Shen X., Li X., Xu C., Wang S. (2007). Mitogen-activated protein kinase OsMPK6 negatively regulates rice disease resistance to bacterial pathogens. Planta.

[196] Cochrane F.C., Davin L.B., Lewis N.G. (2004). The Arabidopsis phenylalanine ammonia lyase gene family: Kinetic characterization of the four PAL isoforms. Phytochemistry.

[197] Ke Y., Deng H., Wang S. (2017). Advances in understanding broad-spectrum resistance to pathogens in rice. Plant J..

[198] Brown J.K.M. (2002). Yield penalties of disease resistance in crops. Curr. Opin. Plant Biol..

[199] Xiao S., Calis O., Patrick E., Zhang G., Charoenwattana P., Muskett P. (2005). The atypical resistance gene, RPW8, recruits components of basal defence for powdery mildew resistance in Arabidopsis. Plant J..

[200] Fukuoka S., Saka N., Koga H., Ono K., Shimizu T., Ebana K., Hayashi N., Takahashi A., Hirochika H., Okuno K. (2009). Loss of function of a proline-containing protein confers durable disease resistance in rice. Science.

[201] Deng Y., Zhai K., Xie Z., Yang D., Zhu X., Liu J., Wang X., Qin P., Yang Y., Zhang G. (2017). Epigenetic regulation of antagonistic receptors confers rice blast resistance with yield balance. Science.

[202] Zhou X., Liao H., Chern M., Yin J., Chen Y., Wang J., Zhu X., Chen Z., Yuan C., Zhao W. (2018). Loss of function of a rice TPR-domain RNA-binding protein confers broad-spectrum disease resistance. Proc. Natl. Acad. Sci. USA.

